# Circulating endothelial cells (CECS) and circulating endothelial progenitor cells (CEPCS) in septic and non-infectious systemic inflammatory response syndrome

**DOI:** 10.1186/2197-425X-3-S1-A307

**Published:** 2015-10-01

**Authors:** S Fernández, P Castro, P Molina, M Palomo, J Aibar, M Díaz-Ricart, JM Nicolás

**Affiliations:** Hospital Clinic of Barcelona, Medical Intensive Care Unit, Barcelona, Spain; Hospital Clinic of Barcelona, Hemotherapy-Hemostasis Department, Barcelona, Spain

## Introduction

Endothelial dysfunction plays a key role in sepsis physiopathology, leading to multiorgan failure and death. Circulating endothelial cells (CECs) and circulating endothelial progenitor cells (CEPCs) may be elevated in septic syndrome as a result of endothelial damage. Previous studies have shown conflicting results and their role in septic syndrome remains unclear [1, 2].

## Objectives

Compare the number of circulating endothelial cells (CECs) and circulating endothelial progenitor cells (CEPCs) in septic and non-infectious systemic inflammatory response syndrome (SIRS) and controls; and evaluate their association with outcome.

## Methods

Blood samples from patients affected with septic syndrome (sepsis, severe sepsis and septic shock) and SIRS were prospectively collected within the first 24 hours and after 96 hours of symptoms onset. Patients with malignancies were excluded. A healthy control group was included (n= 15). CECs (defined as CD45-, CD146+ and CD31+) and CEPCs (defined as CD45-, CD34+ and KDR+) count were determined by flow cytometry and were calculated to obtain their absolute number per 1 mL of whole blood.

## Results

Fifty-seven patients were included in the study: sepsis (n = 20), severe sepsis (n = 10), septic shock (n = 16) and non-infectious SIRS (n = 11). Sixty-five percent were male and median age was 68 years. Most of the patients presented non-surgical pathologies (93%). Patients of all groups presented higher number of CECs and CEPCs than healthy volunteers in baseline (p < 0.001 and 0.034 respectively). A positive correlation was found between CECs and CEPCs count in the first day in all groups (p < 0.001). CECs and CEPCs count decreased significantly in patients with severe sepsis/septic shock at 96 hours (p = 0.034). There were not significant differences between CECs and CEPCs count either between the different groups or survivors and non-survivors. We did not find any correlation between the number of CECs or CEPCs and severity or organ dysfunction at any time point.

## Conclusions

Patients with SIRS of infectious and non-infectious origin present an increase of CECs and CEPCs count. However, different septic syndrome and non-infectious SIRS do not present differences in these count. Finally, CECs and CEPCs count seem not associated with survival or illness severity in these patients.

## Grant Acknowledgment

FIS PI12/00832, Instituto de Salud Carlos II y cofinancaido con Fono Europeo de Desarrollo Regional (FEDER), Unión Europea, Una manera de hacer Europa.
